# Motivations and Barriers to Sharing Biological Samples: A Case Study

**DOI:** 10.3390/jpm3020102

**Published:** 2013-06-06

**Authors:** Stacey Pereira

**Affiliations:** Center for Medical Ethics and Health Policy, Baylor College of Medicine, Houston, TX 77030, USA; E-Mail: spereira@bcm.edu; Tel.: +1-713-798-1012; Fax: +1-713-798-5678

**Keywords:** biobank, genomics, tissue samples

## Abstract

One of the most significant impediments to the current goals of genomic research is the limited availability of high quality biological samples. Despite efforts to increase both the quality and quantity of samples collected, access to such samples remains limited. This may be due, at least in part, to a general reluctance of biobanking professionals, clinicians, and researchers to share biological specimens with others. Ethnographic methods were used in a biobank setting to explore professionals’ perspectives toward and practices of sharing samples. Several motivations and barriers to sharing that may influence research practice were identified. Contrary to existing literature that suggests that professionals are unlikely to share samples with one another, the participants of this study were open to and supportive of sharing samples for research. However, clear communication and effective infrastructure are needed to support the distribution of biobank materials.

## 1. Introduction

The limited availability of high quality biological samples is currently one of the most significant obstacles to genomic research of human health and disease. The difficultly in procuring samples has been referred to as: “the rate-limiting step” for some genomic research [1], “a major roadblock to translational research and personalized medicine” [2], and “the number one roadblock to a cure (for cancer)” [3]. Efforts to increase the quantity and quality of samples collected for genomic research have largely centered around the establishment of biobanks and the implementation of enhanced quality control measures. Though these measures may have resulted in increased collection and improved quality of samples, access to samples remains limited. A recent national survey of cancer researchers showed that many of those surveyed felt that it was difficult to obtain both the number and quality of samples they needed to conduct their research [4]. Likewise, the difficulty in obtaining enough high quality samples to meet current research goals has been bemoaned by those involved with several national research initiatives in the US, including the Cancer Human Biobank (caHUB) of the National Cancer Institute (NCI), and The Cancer Genome Atlas (TCGA), a joint project between the NCI and the National Human Genome Research Institute (NHGRI) [1,5].

This difficulty in obtaining biological samples for research may be due, at least in part, to researchers and other medical professionals’ unwillingness to share samples with one another. A culture of broad data sharing has developed over the past decade within the field of genomics, as reflected in national [6,7] and international [8,9] data sharing policies. However, it seems this practice of sharing has not translated from data to tissue. The reluctance of clinicians and researchers to share samples with one another has been cited in the literature [2,10,11], the popular press [3,12], and in relation to specific research entities [3,5,13,14,15]. Biobanks have been characterized as being largely unwilling to share samples outside of their own institutions [12], and researchers have been accused of working in “silos,” across which samples are rarely shared [2].

Data may be shared more freely than tissues because of the ease of reproducing data, compared to the finite nature of tissue samples. However, even the creation of renewable sources of tissue for research, such as cell lines and xenografts, has not alleviated the problem. Other potential barriers to sharing biological samples include concerns about academic credit and commercial interests. It has been suggested, for example, that clinical researchers are reluctant to share samples with molecular biologists for fear that the latter will receive all the credit for the research, despite the clinicians’ long-term work of carefully collecting and annotating the samples [10]. Andrews [11] highlights two scenarios in which commercial interests might impose a barrier to sharing, both based on anecdotal cases. In the first, a geneticist reported that he had tried to undertake a study using leftover tissue in his hospital, but found that the department had sold the tissue to a biotechnology company. In the second, several autism researchers, who reported that individually they did not have enough samples to carry out their research, declined to share with one another because they each wanted to be able to patent the gene associated with the disorder.

Despite being identified as an obstacle to obtaining samples for important research, the purported reluctance of biobank professionals and researchers to share biological samples with one another has not yet been directly studied. Additionally, the equally important topic of what motivates biobank professionals to share samples has been overlooked. Here, the findings of a qualitative case study of professionals’ motivations and barriers to sharing biological samples are reported.

## 2. Methods

### 2.1. Data Collection

This study was conducted using ethnographic methods in the context of a newly formed, statewide cancer biobank, with which the author is a collaborator. Participant observation was used to explore in-depth the practices and perspectives of different professionals involved in the collection, storage, and distribution of biological samples and associated data. Participant observation is a method used extensively in ethnographic and anthropological research and is characterized by the immersion of the researcher as both observer and participant in the normal, everyday practices of the group being studied, usually for an extended period of time [16]. This approach is especially well suited to exploratory research such as this, where there is little to no existing data on the topic, and allows the researcher to gain insight into the participants’ point of view without imposing predetermined themes. This method also allows the researcher to obtain data that may not be accessible via other means, such as observations of what participants are actually doing as opposed to data on what they say they do, and data on practices and perspectives over a span of time.

Data were collected through observation and active participation in biobank activities, such as bi-monthly project meetings, deliberations of the governance committee, working group discussions, and annual planning and strategy retreats over the course of 14 months (September 2011–November 2012). Detailed field notes were taken throughout participant observation [16].

### 2.2. Research Site and Participants

The site for this study was a statewide cancer biobank that collects and distributes tumor and normal biological samples and derivatives, extracted germline and somatic DNA, and associated clinical and genomic data. It represents a collaborative effort between many different professionals across a range of biomedical subspecialties from both academic institutions and for-profit companies. It operates in a federated, virtual manner, in which contributors collect and maintain their own samples at their respective sites, but the entire collection is maintained virtually and managed by one centralized governance committee. The purpose of the governance committee is to review requests for materials and allocate available human samples and derivatives, as well as data to researchers for scientifically valid investigations. Membership of the committee comprises a broad representation, including a bioethicist, a biostatistician, members from all contributing sites, other scientific experts outside the biobank, and a community representative.

All professionals engaged either directly or indirectly with this biobank were invited to participate in this project. This includes all professionals who participated in any of the biobank meetings and events described above, as well as professionals who were less directly involved in the biobank yet had an influence on the collection, banking, and/or distribution of samples, such as hospital pathologists. All potential participants were informed that we were undertaking an ethnographic study using participant observation methods within the biobank to explore practices and perspectives toward the collection, banking, and research use of biological samples. All professionals were given an opportunity to decline participation; no one declined. Participants were 64 professionals across a range of professional groups, including surgeons, medical oncologists, tissue advocates, pathologists, genome scientists, informaticians, and members of the biobank’s governance committee. This study was approved by the Baylor College of Medicine Institutional Review Board.

In this paper, the term “participants” refers to the professionals who took part in this study, and the term “contributor” refers to professionals who collect and contribute biological samples to the biobank.

### 2.3. Data Analysis

Data were qualitatively analyzed using thematic content analysis [17]. The overall goal of the analysis was to identify motivations and barriers to sharing biological samples. Motivations are defined as any reason or rationale behind a professional’s desire to share samples. Barriers are perspectives that deter or hinder sharing. Field notes were coded for practices and discussions related to the sharing and/or retention of samples, explicitly stated motivations and barriers to sharing samples, and values and perspectives that were identified as potentially related to sharing samples. Data were then analyzed for recurring themes related to sharing and retention of samples and categorized observations into groups by theme.

## 3. Results

### 3.1. Barriers to Sharing Biological Samples for Research

Qualitative analysis of participant observation field notes revealed three major barriers to sharing biological samples among professionals, which are presented below in order of frequency of occurrence. The most common barrier was the desire to ensure that the collected samples are put to optimal use in studies with high scientific merit and potential impact, rather than allow samples to be used in research that is perceived to be less promising or less important. For example, in one instance, the biobank’s governance committee denied a request for samples of a type that was considered to be a more precious resource because the requestor’s proposed study was exploratory. Committee members felt that, as exploratory research, that particular study did not justify the use of those more valuable samples. Instead, the committee asked the requestor to carry out the exploratory research using less valuable samples, with the option of requesting the more valuable samples later if the exploratory research proved promising.

This desire to ensure optimal use of the samples was a barrier to sharing not only in the day-to-day operations of the biobank, but also in the development of official biobank policies. When developing a process for evaluating requests for sample distribution, several proposals were made that would restrict access but ensure optimal use. For example, several biobank professionals advocated for scientific review of proposed research using a specific scoring system akin to the review of grant proposals. Others took this proposition further, suggesting that requests be ranked for potential approval, or that the minimum score necessary for approval be set higher for materials considered to be of a comparatively higher value.

Similarly, another biobank professional suggested that samples be “embargoed” once a portion of those samples had been distributed to a researcher in case that recipient needed additional amounts of material in order to complete the project. This restriction on the use of the remaining sample, though presenting a barrier to sharing and other researchers’ access to the biobank materials, would facilitate the successful completion of research for which biobank materials have been given, and, thus, would help to ensure optimal use of the samples after distribution.

Another professional focused more on the use of the samples overall in terms of optimal utilization of the entire collection, stressing the importance of tracking the types of projects for which samples are given and avoiding distribution of samples for use in several similar projects. This might help to ensure use of biobank materials for varied projects and prevent duplication of research efforts with the materials. Similarly, in another example, a governance committee member felt that single-gene studies, as opposed to genome-wide analyses, were not the best use of biobank materials and pointed out that approving multiple single-gene studies would reduce the overall impact of the collection. One biobank professional summed up the trade-off many were considering between conserving limited materials and facilitating high quality research when he stated that although samples are a “scarce resource,” he was “happy to send them for a *good* project”.

A second barrier to sharing samples was related to the primary obligation to provide appropriate clinical care to patients. This observed barrier was specific to pathologists who were not directly involved in the biobank. Tissue samples that were collected for the biobank were often obtained from pathology laboratories, where the entirety of the excised tissue is taken for analysis after the patient’s surgery. In several cases, a negotiation would then take place between the biobank staff and the pathologist regarding how much of the excised tissue should be submitted to the biobank versus how much should be retained in pathology records for potential re-evaluation of the patient’s clinical diagnosis in the future. Here we can see the value of wanting to ensure appropriate clinical care as a potential barrier to sharing samples for research.

Finally, confusion over “ownership” of the samples seemed to create a barrier to sharing in a few circumstances. The question of who owns biological specimens has generated much legal and ethical debate [18,19,20,21]. Empirical studies have shown that there is much confusion about who owns samples and what “ownership” actually entails [22,23,24]. Concerns regarding appropriate ownership of samples seemed to create a barrier to sharing from the early stages of this biobank. In one case, governance committee members were hesitant to review a request for samples because it was unclear to whom those samples belonged. Confusion over whether it was the biobank or an individual researcher who “owned” the samples led to uncertainty about whether they could be distributed as the committee saw fit. This issue was ultimately managed through explicit agreement of all parties involved in the biobank that the governance committee had full authority to decide how samples would be distributed, irrespective of who owned the sample.

### 3.2. Motivations for Sharing Biological Samples for Research

Analysis of field notes also revealed several motivations for sharing samples. The most commonly observed motivation was the desire for collected samples to be used in research, as opposed to being stored in freezers indefinitely. One contributor made a point of stressing the importance of using the samples in research several times over the course of planning meetings by asking what the purpose of spending time and effort to collect samples would be otherwise. She was also vocal about not wanting to contribute to any banking projects where samples were not regularly shared with other researchers. In a similar vein, another professional advocated for requiring recipients of biobank materials to report updates on their projects so that the group could be sure that the samples were used for research after distribution. A common goal articulated throughout the project was that the biobank should be empty, and that a bank full of tissues at the end of the initial grant period would signify failure of the project.

A second motivation for sharing samples was the desire to make samples more easily accessible for research. One professional specifically advocated keeping the requirements and responsibilities of recipients of biobank materials to a minimum: “the idea is to lower the barrier; this is about changing the model.” At another point, the same professional suggested that the impetus for this particular biobank was the fact that tissues are so difficult to obtain for research.

The desire to increase the value of banked materials with associated genomic data served as a third motivation to share samples with other researchers. For example, in one biobank meeting, a contributing member supported prioritizing sending samples for use in a large, national research project, but only if the project would return genomic results to the biobank. The rationale was that samples with full genome characterization would be more valuable. Likewise, another professional urged the group to send samples for genomic analysis in order to produce sequence data for the biobank.

A more self-interested motivation for sharing samples was the desire to increase the visibility of the institution and the biobanking group in the research community. One biobank professional advocated strongly for sharing samples with a national cancer genome project, stressing that being a known contributor to this project would be advantageous to the biobank as a whole. Additionally, many felt that increased visibility of the biobank would lead to increased utility to the research community, as it would help make researchers aware of the availability of samples.

## 4. Discussion

Participants in this study were very supportive of research and were motivated to make samples widely available and easily accessible. The barriers to sharing samples tended to center around issues and concerns related to ensuring proper management of the biobank’s resources.

As discussed, the literature and specific research initiatives decry a general lack of available samples. Common explanations for why samples are not more widely available tend to depict clinicians, biobanking professionals, and researchers as self-interested for both academic and commercial pursuits, to the detriment of research. This case study, however, suggests that biobanking professionals are actually quite open to sharing samples. The professionals observed showed considerable interest in advancing research and a generally altruistic perspective toward sharing samples and making materials accessible to the research community. Any hesitation to share samples was more about ensuring the best possible use of the biobank’s materials than personal academic or commercial pursuits. To the extent that these biobank professionals expressed self-interest, it was in the form of a general interest in increasing the visibility and value of the bank and ultimately served as a motivation, rather than a barrier, to sharing.

More often, barriers to sharing samples reflected poor communication or lack of infrastructure, rather than self-interest. For example, requesting researchers often were not clear in filling out their request forms, or failed to supply important information that was necessary for the governance committee to review the request. In other cases, there was confusion regarding institutional policies that might affect how samples were shared, such as policies about licensing agreements, which in turn slowed the distribution process considerably. These issues played a significant role in disrupting distribution of materials and highlight the importance of building an effective infrastructure with clear communication processes.

A limitation of this study is its focus on one biobank, which may not be representative of others. In choosing to use ethnographic methods, we emphasized depth over breadth in the research design. This approach facilitated the capture the motivations and barriers to sharing samples as they were occurring, which may not have been identified using other methods such as surveys and other self-report measures, where investigators would be asked to respond to a set of predetermined items. This case study may serve as a basis for further research that spans multiple biobanks. A second limitation of this study is its focus on those who are already directly or indirectly involved in the biobank. Future analyses should focus on those who are aware of or have been invited to submit samples to the biobank, but have declined; data on the reasons for declining to contribute to the biobank will be especially important in developing a fuller understanding of the barriers to sharing biological samples. The author is an active collaborator with the biobank. Although active participation is common in ethnographic research, this relationship with the biobank may have influenced some of the discussions around sample sharing. Finally, although the use of participant observation is thought to reduce the potential for reactivity [16], it is possible that participants altered their behavior to create a more favorable impression because they were aware they were being observed. 

Future research should explore whether the observations in this case study can be generalized to other projects and professionals. There are trends that suggest a difference between professional groups in their attitudes toward sample sharing, but additional data is necessary in order to fully explore whether these differences reflect individual values or the professional values of different subspecialties.

## 5. Conclusion

Understanding the motivations and barriers of professionals to sharing biological samples is the first step toward addressing the problem of limited availability. Although it is often assumed that biobank professionals are reluctant to share samples because it is not in their self-interest, this is not consistent with the results of this study. The biobanking professionals observed were generally open to sharing samples with others and were only reluctant to share if doing so might not result in the best possible use of biobank materials. Obstacles to sharing samples were more administrative than value-laden. Establishing clear communication practices and building effective infrastructure thus seem to be the most important ways to support biobank distribution.
